# Cardiovascular Risk of NSAIDs: Time to Translate Knowledge into Practice

**DOI:** 10.1371/journal.pmed.1001389

**Published:** 2013-02-12

**Authors:** K. Srinath Reddy, Ambuj Roy

**Affiliations:** 1 Public Health Foundation of India, ISID Campus, Vasant Kunj, New Delhi, India; 2 Department of Cardiology, Cardio Thoracic Centre, All India Institute of Medical Sciences, Ansari Nagar, New Delhi, India

## Abstract

K. Srinath Reddy and Ambuj Roy discuss the Research Article by Patricia McGettigan and David Henry about the continued use of NSAIDs associated with increased risk of cardiovascular disease, including the mechanisms of increased risk, probable reasons for ongoing use, and next steps.

**Linked Research Article**

This Perspective discusses the following new study published in *PLOS Medicine*:

McGettigan P, Henry D (2013) Use of Non-Steroidal Anti-Inflammatory Drugs That Elevate Cardiovascular Risk: An Examination of Sales and Essential Medicines Lists in Low-, Middle-, and High-Income Countries. PLoS Med 10(2): e1001388. doi:10.1371/journal.pmed.1001388

Patricia McGettigan and David Henry find that, although some non-steroidal anti-inflammatory drugs (NSAIDs) such as diclofenac are known to increase cardiovascular risk, diclofenac is included on 74 countries’ essential medicine lists and was the most commonly used NSAID in the 15 countries they evaluated.

Clinical use of non-steroidal anti-inflammatory drugs (NSAIDs) carries cardiovascular risk, which acquires implications for public health against the backdrop of rising chronic disease rates in low- and middle-income countries (LMICs). New evidence from an international study conducted by Patricia McGettigan and David Henry and published this week in *PLOS Medicine* [1] sheds light on how emerging evidence about NSAID risk is poorly translated into practice and sales in countries around the world, raising questions about the use and promotion of potentially harmful drugs.

NSAIDs are extensively prescribed for pain management in patients with osteoarthritis and several other painful conditions. The large number of drugs in this group is broadly divided into nonselective cyclo-oxygenase (COX) inhibitors and selective COX inhibitors. This classification is based on the selective inhibition of COX-2 enzyme, which is primarily responsible for the generation of inflammatory mediators. The emergence of selective COX-2 inhibitors in the 1990s was widely welcomed by physicians, as these drugs were expected to reduce the adverse gastrointestinal effects associated with inhibition of COX-1. However, the enthusiasm evaporated when it was discovered that rofecoxib (Vioxx), an early and aggressively marketed molecule of this drug class, increased the risk of serious cardiovascular events [2],[3]. Subsequently, several systematic reviews and meta-analyses showed that other NSAIDs too were associated with adverse cardiovascular events [4]–[6].

The adverse cardiovascular profile of NSAIDs includes risk of atherothrombotic events like myocardial infarction (MI) and stroke, which can be fatal. The increased cardiovascular risk has been observed both in people with a prior high risk of cardiovascular disease and in previously healthy individuals [7], and this risk appears to be dose dependent [8]. What is intriguing, however, is that the increase in cardiovascular risk has been variable with the different molecules. Apart from rofecoxib, diclofenac is the agent most associated with an increased risk of cardiovascular events: a 40%–60% higher relative risk of serious cardiovascular events, compared to non-use of NSAIDs, has been reported [4]–[6],[9]. This is a rate equivalent to or possibly higher than that of rofecoxib, now withdrawn from the market. In contrast, another traditional NSAID, naproxen, has been found to be relatively benign, with a cardiovascular risk that was observed to be neutral or much lower than that of diclofenac [4]–[6],[9].

The reason for this variability in cardiovascular risk among the non-selective NSAIDs is not completely understood, but mechanistic research suggests it could be related to the extent of COX-2 inhibition by drugs that do not block COX-1 completely [8]. The higher the level of COX-2 inhibition and the lower the level of COX-1 inhibition, the greater appears to be the risk of thrombotic cardiovascular events like fatal or non-fatal MI and stroke. This probably explains the low cardiovascular risk of naproxen, which completely blocks COX-1 and thus has anti-platelet effects that reduce cardiovascular events. When COX-1 inhibition is incomplete (<95%), enough thromboxane A2 (TxA_2_) is generated for platelet activation [8]. The inhibition of COX-2 reduces the generation of vaso-protective prostacyclin (PGI_2_), a prostaglandin that guards against thrombogenesis, atherogenesis, and high blood pressure [10]. The inhibition of COX-2, coupled with an incomplete inhibition of COX-1, provides a potent thrombogenic stimulus by altering the PGI_2_-TxA_2_ balance. While both diclofenac and naproxen are non-selective, the differences in the COX-1 and COX-2 inhibition each drug achieves may explain the variability in their cardiovascular risk profiles.

## What Did the Authors Find?

McGettigan and Henry report new evidence regarding the use of NSAIDs in 15 countries representing high-, medium-, and low-income countries. They reviewed published evidence regarding the cardiovascular risk profiles of different NSAIDs, confirming that diclofenac is associated with a substantially higher cardiovascular risk than naproxen. Using IMS Health 2011 data, they found that diclofenac had a median of 3-fold higher sales (or prescribing, in the case of England and Canada) than naproxen in the 15 countries studied. The preference for diclofenac over naproxen was seen across high-, middle-, and low-income countries, despite the fact that both drugs have been available in generic form for several years and the information related to their comparative cardiovascular risk profile—where risks associated with diclofenac far outweighs that of naproxen—has been known for nearly a decade. They observe that diclofenac continues to figure in the essential medicines lists (EMLs) of 74 countries, while naproxen features in only 27. Their results are striking, and suggest that immediate action is warranted.

It is worth considering how national EMLs are established. Countries develop their national EMLs by setting up committees comprising national experts, usually linked to government-run medical institutions [11], and their recommendations vary in quality. The list prepared by the World Health Organization (WHO) has influence on the national lists but does not have overriding power [12]. Although the WHO EML contains neither diclofenac nor naproxen, McGettigan and Henry reasonably question why WHO has not provided information on the safety of these drugs, which could inform and influence national EMLs.

India, the second most populous country and not included in the 15 countries McGettigan and Henry studied, exemplifies this disconnect between available evidence versus recommendations and practice. Diclofenac features in India's current National List of Essential Medicines, while naproxen does not. In 2008, the sales of diclofenac were 11.3 times higher than naproxen in financial terms and 9.4 times higher in terms of the number of tablets sold (unpublished data; extracted from Intercontinental Marketing Services Health Database, India, 2008).

## How Can Such Non-Evidence–Based Practice Be Addressed?

It is mainly the responsibility of national health agencies and drug regulatory authorities to strongly caution against the adverse cardiovascular effects of diclofenac and other NSAIDs with poor risk profiles and/or mandate their withdrawal from the market. We believe there is a strong case for removing diclofenac from national EMLs, and that WHO should explain not just what should be included in the EML, but also explain why other NSAIDs (such as diclofenac) are not being included, making the risk estimate explicit. The dangers are especially high in countries where over-the-counter sale of diclofenac is permitted.

At the same time, practicing physicians have a duty to regularly update their knowledge on the drugs they frequently prescribe. While there is usually a time lag between scientific publications and translation of that knowledge into improved practice patterns, the internet should hasten the diffusion of knowledge. Cardiologists and neurologists too should play a greater role by becoming better informed of the adverse effects, disseminating evidence-based recommendations on the risks associated with different NSAIDs to other physicians, and strongly advocating for stricter regulation of NSAIDs with a harmful cardiovascular profile.

LMICs are currently experiencing a rapid health transition with escalating rates of cardiovascular mortality [13],[14]. The dangers of retaining potentially harmful drugs on EMLs are especially high in these countries, where standard treatment guidelines are scarce and continuing education of physicians is usually not mandated. It is not just the case of diclofenac versus naproxen that is at stake. It is the broader challenge of ensuring that everyone responsible for the safety of patients makes informed decisions in an appropriate and timely manner. If we do not collectively rise to that challenge, no NSAID can relieve the pain of that failure.

## Abbreviations

COX: cyclo-oxygenase
EML: essential medicines list
LMIC: low- and middle-income country
MI: myocardial infarction
NSAID: non-steroidal anti-inflammatory drug
WHO: World Health Organization
